# Supplementary material for: Dietary rescue of adult behavioral deficits in the Fmr1 knockout mouse
Source: PLoS One. 2022 Jan 28;17(1):e0262916. doi: 10.1371/journal.pone.0262916 (PMC8797197; doi:10.1371/journal.pone.0262916)
Supplement: S1 Table — (DOCX) [file pone.0262916.s002.docx]

**S1 Table. ANOVA Results for Post-Weaning Paradigm.** * = denotes significant interactions with within-subjects variable. See Results section for further information

|  |  | ANOVA Results | | | Post-Hoc Results |
| --- | --- | --- | --- | --- | --- |
|  |  | Genotype | Diet | Interaction |  |
| Elevated Plus Maze | Distance Moved | F(1, 76) = 1.57, p = 0.21 | F(2, 76) = 0.32, p = 0.74 | F(2, 76) = 2.75, p = 0.07 |  |
|  | Velocity | F(1, 76) = 0.88, p = 0.35 | F(2, 76) = 0.99, p = 0.38 | F(2, 76) = 2.96, p = 0.06 |  |
|  | % Time in Open Arms | F(1, 76) = 0.003, p = 0.96 | F(2,76) = 2.03, p = 0.14 | F(2, 76) = 1.54, p = 0.22 |  |
| Sensorimotor Gating Assessment | % Inhibition | F(1, 81) = 6.15, p = 0.02 | F(2, 81) = 0.05, p = 0.95 | F(2, 81) = 3.70, p = 0.03 | Standard WT vs Standard KO: p = 0.002; Standard WT vs Control Fat KO, p = 0.05  Standard WT vs Omega-3 KO; p = 0.04 |
|  | Startle Responding | F(1, 81) = 1.72, p = 0.19* | F(2, 81) = 0.42, p = 0.66 | F(2, 81) = 0.65, p = 0.53* |  |
| Delay Fear Conditioning | Acquisition | F(1, 80) = 1.06, p = 0.31 | F(2, 80) = 11.38, p = 0.0001* | F(2, 80) = 3.18, p = 0.05* | At ITI 1, Tone 2 and ITI 2 – Standard WT vs All Groups, p < 0.05 |
|  | Contextual Fear Conditioning | F(1, 80) = 1.22, p = 0.27 | F(2, 80) = 11.07, p = 0.0001 | F(2, 80) = 1.37, p = 0.21 | Standard vs Control Fat Diet, p < 0.05; Standard vs Omega-3 Diet, p < 0.05 |
|  | Cued Recall | F(1, 80) = 1.0, p = 0.76 | F(2, 80) = 9.88, p = 0.0001 | F(2, 80) = 0.39 p = 0.68 | Standard vs Control Fat Diet, p < 0.05; Standard vs Omega-3 Diet, p < 0.05 |
| PCR | BDNF | F(1, 29) = 0.04, p = 0.84 | F(2, 29) = 0.39, p = 0.68 | F(2, 29) = 1.07 p = 0.36 |  |
|  | IL-1β | F(1, 29) = 0.95, p = 0.34 | F(2, 29) = 1.54, p = 0.23 | F(2, 29) = 0.14, p = 0.87 |  |
|  | IL-6 | F(1, 29) = 4.57, p = 0.04 | F(2, 29) = 4.29, p = 0.02 | F(2, 29) = 0.46, p = 0.63 | Standard vs Omega-3 Diet, p < 0.05 |
|  | TNF-α | F(1, 29) = 7.91, p = 0.01 | F(2, 29) = 0.40, p = 0.67 | F(2, 29) = 0.67, p = 0.52 |  |
