# Supplementary material for: Dietary rescue of adult behavioral deficits in the Fmr1 knockout mouse
Source: PLoS One. 2022 Jan 28;17(1):e0262916. doi: 10.1371/journal.pone.0262916 (PMC8797197; doi:10.1371/journal.pone.0262916)
Supplement: S2 Table — (DOCX) [file pone.0262916.s003.docx]

**S2 Table. ANOVA Results for Perinatal Paradigm.** * = denotes significant interactions with within-subjects variable. See Results section for further information

|  |  | ANOVA Results | | | Post-Hoc Results |
| --- | --- | --- | --- | --- | --- |
|  |  | Genotype | Diet | Interaction |  |
| Elevated Plus Maze | Distance Moved | F(1, 84) = 21.04, p = 0.001 | F(2, 84) = 4.26, p = 0.02 | F(2, 84) = 3.35, p = 0.04 | Control Fat KO vs Standard WT, p = 0.02; Control Fat WT vs Standard WT, p = 0.004; Omega-3 WT vs Standard WT p = 0.01; Standard WT vs Omega-3 KO, p = 0.62 |
|  | Velocity | F(1, 84) = 14.41, p = 0.001 | F(2, 84) = 7.52, p = 0.001 | F(2, 84) = 1.21, p = 0.30 | Standard vs Omega-3 Diet, p < 0.05; Control Fat vs Omega-3 Diet, p < 0.05 |
|  | % Time in Open Arms | F(1, 84) = 7.91, p = 0.01 | F(2, 84) = 8.01, p = 0.001 | F(2, 84) = 2.10, p = 0.13 | Standard vs Control Fat Diet, p < 0.05 |
| Sensorimotor Gating Assessment | % Inhibition | F(1, 84) = 7.74, p = 0.01* | F(2, 84) = 5.55, p = 0.01 | F(2, 84) = 2.89, p = 0.06 | Standard vs Control Fat Diet, p < 0.05 |
|  | Startle Responding | F(1, 84) = 0.02, p = 0.88* | F(2, 84) = 4.01, p = 0.02* | F(2, 84) = 0.28, p = 0.76* |  |
| Delay Fear Conditioning | Acquisition | F(1, 90) = 3.40, p = 0.07* | F(2, 90) = 5.08, p = 0.01* | F(2, 90) = 2.77, p = 0.07 | Standard vs Control Fat Diet, p < 0.05; Standard vs Omega-3 Diet, p < 0.05 |
|  | Contextual Fear Conditioning | F(1, 90) = 0.47, p = 0.50 | F(2, 90) = 0.87, p = 0.42 | F(2, 90) = 0.77, p = 0.47 |  |
|  | Cued Recall | F(1, 90) = 10.30, p = 0.002* | F(2, 90) = 0.66, p = 0.52 | F(2, 90) = 0.60, p = 0.52 |  |
| PCR | BDNF | F(1, 30) = 0.007, p = 0.93 | F(2, 30) = 7.38, p = 0.003 | F(2, 30) = 0.33, p = 0.72 | Standard vs Omega-3 Diet, p = 0.004; Standard vs Control Fat Diet, p < 0.0001 |
|  | IL-1β | F(1, 30) = 0.42, p = 0.52 | F(2, 30) = 8.03, p = 0.002 | F(2, 30) = 0.16, p = 0.85 | Standard vs Omega-3 Diet, p = 0.001; Standard vs Control Fat Diet, p = 0.002 |
|  | IL-6 | F(1, 30) = 0.04, p = 0.84 | F(2, 30) = 2.62, p = 0.09 | F(2, 30) = 0.02, p = 0.98 |  |
|  | TNF-α | F(1, 30) = 0.38, p = 0.54 | F(2, 30) = 2.16, p = 0.13 | F(2, 30) = 0.11, p = 0.89 |  |
