# Supplementary material for: Dietary rescue of adult behavioral deficits in the Fmr1 knockout mouse
Source: PLoS One. 2022 Jan 28;17(1):e0262916. doi: 10.1371/journal.pone.0262916 (PMC8797197; doi:10.1371/journal.pone.0262916)
Supplement: S3 Table — (DOCX) [file pone.0262916.s004.docx]

**S3 Table. Cytokine Associations with Behavior for Both Paradigms.** Variables Measured: 1) Acquisition Phase Response to Tone; 2) Freezing during Contextual Phase; 3) Freezing to Tone During Cued Recall Phase; 4) Distance Moved in EPM; 5) Velocity during EPM. * = p < 0.05; ** = p < 0.01.

|  | Post-Weaning Paradigm Measures | | | | | Perinatal Paradigm Measures | | | | |
| --- | --- | --- | --- | --- | --- | --- | --- | --- | --- | --- |
|  | 1 | 2 | 3 | 4 | 5 | 1 | 2 | 3 | 4 | 5 |
| BDNF | -.13 | -.11 | .13 | -.06 | -.04 | **-.34*** | -.25 | -.10 | .03 | -.07 |
| IL-1β | .20 | .23 | .09 | .15 | .17 | **-.35*** | -.27 | -.07 | .11 | .04 |
| IL-6 | **.40*** | **.43**** | .12 | -.24 | -.22 | -.27 | -.09 | -.07 | .08 | .10 |
| TNFα | .04 | .32 | .04 | -.33 | **-.40*** | -.20 | -.17 | -.04 | -.008 | -.12 |
