# Supplementary material for: Dietary rescue of adult behavioral deficits in the Fmr1 knockout mouse
Source: PLoS One. 2022 Jan 28;17(1):e0262916. doi: 10.1371/journal.pone.0262916 (PMC8797197; doi:10.1371/journal.pone.0262916)
Supplement: Attachment — Submitted filename: Response to Reviewers.docx [file pone.0262916.s006.docx]

Reviewer #1: Re: PONE-D-21-20764R1

Nolan et al describe the effects of dietary rescue of omega-3 fatty acids at two different time points in the Fmr1 KO mouse, which they have resubmitted. It is of interest, but there still a couple of remaining issues with the communication of the findings. They have addressed the remainder of my comments.

Methods- new text at the end of the first section- ‘Only male mice were used for the present studies, as previous work from our lab did not 129 find strong evidence of a phenotype in many of the tasks included here’- do the authors mean to add ‘…in females’ here? Otherwise, please clarify.

We addressed this comment by noting that we did not find strong evidence of a phenotype in female mutants. Thank you for catching this exclusion!

Results- For the startle and PPI results, second paragraph, ‘Diet did not significantly impact percent inhibition alone’- which diet timeframe? This detail is still missing

This timeframe has been clarifying in the appropriate text.

Discussion- Paragraph 4- The lack of effect of the mutation on social behavior and stereotypy is fleetingly mentioned- should briefly place this in context of what is expected.

We expanded this portion of the discussion to note that based on previous findings in both our labs and others that this phenotype is not typically noted, though is more common in rat models.

Finally, the last paragraph should present more awareness of how much needs to be done before this can be translated clinically.

We expanded this paragraph to note that though many treatments have been posited by preclinical research, their efficacy in humans is still not clear and this treatment deserves the same scrutiny despite the promising results demonstrated here.
